# Fitness for work on atypical schedules in workers with psycho-affective disorders

**DOI:** 10.1192/j.eurpsy.2023.1014

**Published:** 2023-07-19

**Authors:** W. Ayed, D. Brahim, I. Youssef, N. Mechergui, H. Ben Said, M. Mersni, S. Ernez, G. Bahri, N. Ladhari

**Affiliations:** Occupational pathology and fitness for work, Charles Nicolle Hospital, Tunis, Tunisia

## Abstract

**Introduction:**

Work on atypical schedules could lead to alertness and sleep disorders, which makes people with psychiatric pathologies more likely to exacerbate their illness.

**Objectives:**

To study the impact of psychoaffective diseases on the fitness for night or/and shiftwork

**Methods:**

A descriptive cross-sectional study was conducted with patients with psychoaffective disorders working atypical hours who have consulted the Occupational Medicine Department of the Charles Nicolle Hospital for statements of medical fitness. The study period was six years from January 2016 to June 2022.

**Results:**

Among 224 employees who had shift/night work , 32.1% (n=76) had psycho-affective disorders. The average age was 43.32±8.64 years. The sex ratio (M/F) was 0.46. The average professional seniority was 17.35±9.17 years. The most represented sectors were: health (56%), the electronics industry (5%), finance (5%) and the plastics industry (5%). The most occupied jobs were: nurses (21%), blue collar workers (20%), senior techniciens (20%) and security guards (8%). Psychiatric pathologies were represented by anxiety disorders (80%), psychoses (8%), schizophrenia (8%) and bipolar disorders (4%). The consultants were on medication in 88% of cases. Antidepressants were prescribed in 75% of cases, followed by anxiolytics (54%), antipsychotics (22%) and thymoregulators (4%). Concerning the medical fitness for work of the patients, a definitive eviction from shift/night work was indicated in 56% of cases.

**Conclusions:**

A medical assessment of the fitness to work on atypical schedules for workers with psychiatric disorders is required, in particular, during the employment medical examination

**Disclosure of Interest:**

None Declared

